# Two Hybrid Histidine Kinases Involved in the Ethylene Regulation of the Mycelial Growth and Postharvest Fruiting Body Maturation and Senescence of Agaricus bisporus

**DOI:** 10.1128/spectrum.02411-22

**Published:** 2022-09-20

**Authors:** Chaohui Zhang, Di Shang, Yan Zhang, Xiyang Gao, Dehai Liu, Yuqian Gao, Yanan Li, Yuancheng Qi, Liyou Qiu

**Affiliations:** a College of Life Sciences, Henan Agricultural University, Key Laboratory of Enzyme Engineering of Agricultural Microbiology, Ministry of Agriculture and Rural Affairs, Zhengzhou, China; b College of Life Science and Technology, Henan Institute of Science and Technology, Xinxiang, China; c Institute of Biology Co., Ltd., Henan Academy of Science, Zhengzhou, China; University of Michigan

**Keywords:** hybrid histidine kinase, ethylene receptor, basidiomycetes, ethylene-binding domain, ethylene-binding activity, maturation- and senescence-related genes

## Abstract

Ethylene regulates mycelial growth, primordium formation, and postharvest mushroom maturation and senescence in the white button mushroom, Agaricus bisporus. However, it remains unknown how ethylene is detected by the mushroom. In this study, we found that two hybrid histidine kinases in the mushroom, designated *Ab*ETR1 and *Ab*ETR2, showed domain structures similar to those of plant ethylene receptors. The transmembrane helices of *Ab*ETR1 and *Ab*ETR2 were expressed in yeast cells and showed ethylene-binding activities. Mushroom strains with downregulated expressions of *Ab*ETR1 and *Ab*ETR2 showed reduced sensitivity to the ethylene inhibition of mycelial growth, ethylene regulation of their own synthesis, postharvest mushroom maturation, and senescence and expression of maturation- and senescence-related genes. Therefore, *Ab*ETR1 and *Ab*ETR2 are expected to be biologically functional ethylene receptors and exhibit a different mode of action from that of the receptors of plants. Here, we fill gaps in the knowledge pertaining to higher fungus ethylene receptors, discover a novel mode of action of ethylene receptors, confirm ethylene as a novel fungal hormone, and provide a facilitated approach for preventing the maturation and senescence of postharvest button mushrooms.

**IMPORTANCE** Ethylene regulates diverse physiological activities in bacteria, cyanobacteria, fungi, and plants, but how to perceive ethylene by fungi only remains unknown. In this study, we identify two biologically functional ethylene receptors in the basidiomycete fungus Agaricus bisporus, which fills the gaps of deficient fungal ethylene receptors. Furthermore, we found that decreased expression of the ethylene receptors facilitates preventing the maturation and senescence of postharvest button mushrooms, indicating that the two fungal ethylene receptors positively regulate the ethylene response, in contrast to that in plants.

## INTRODUCTION

Ethylene is an important plant hormone that regulates seed germination, seedling growth and development, fruit ripening, organ maturation and shedding, and stress responses (1, 2). Many bacteria and fungi also synthesize ethylene, including at least 88 yeasts and molds, such as Saccharomyces cerevisiae, *Schizosaccharomyces*, Cryptococcus albidus, *Rhizopus*, Aspergillus, *Penicillium*, mycorrhizal fungi, edible-medicinal macrofungi, phytopathogenic fungi, and human- and animal-pathogenic fungi (3). Plants synthesize ethylene via the 1-aminocyclopropane-1-carboxylate (ACC) pathway, while fungi synthesize ethylene via the 2-keto-4-methylthiobutyric acid (KMBA) pathway and the ACC pathway (4). Ethylene influences fungal growth, metabolism, and interactions with plants (5, 6). However, how fungi perceive and respond to ethylene is still unknown.

The perception of and response to ethylene in embryophytes, charophytes, and chlorophytes occur through an ethylene signal transduction pathway consisting of ethylene receptors (ETRs), ethylene signaling molecules, and ethylene-responsive transcription factors (7). There are five ethylene receptors in the model plant Arabidopsis thaliana that mediate its response to ethylene in a negatively regulated manner (8–10). In contrast, incomplete ethylene signal transduction pathways are probably present in the genomes of glaucophytes, rhodophytes, and cyanobacteria (11); specifically, ethylene receptors found in *Synechocystis* sp. strain PCC 6803 (12, 13) and *Geitlerinema* sp. strain PCC 7105 (14) can sense and respond to ethylene. Several putative ethylene receptors are present in early diverging fungi, Mesomycetozoa, Amoebozoa, diatoms, Zooxanthellae, and Chromerida (15, 16), but whether they sense and respond to ethylene is unknown. Interestingly, although Neurospora crassa and Rhizopus stolonifer have been found to show [^14^C] ethylene-binding activity (17), the ethylene receptors of higher fungi, including ascomycetes and basidiomycetes, have not been reported.

Plant ethylene receptors originate from a bacterial two-component signal transduction system composed of a homodimeric histidine kinase (HK) and a response regulator (RR). Two-component signal transduction systems exist in microorganisms and plants and are not found in humans and metazoans (18). The two-component signal transduction system evolved into a hybrid histidine kinase system with the fusion of HK and RR in most eukaryotes. The plant ethylene receptors are transmembrane proteins localized on the endoplasmic reticulum membrane showing a similar overall modular structure, as exemplified by the representative ethylene receptor ETR1 in *A. thaliana* (AtETR1), which is composed of an ethylene-binding domain (EBD; also referred to as a transmembrane sensor domain [TMD]), GAF domain, histidine kinase domain, and receiver domain (19–21). Plants have a family of ethylene receptors, which can be divided into two subfamilies on the basis of their protein sequences and structural features. Subfamily I receptors have three transmembrane regions at their N-terminal ends and a conserved histidine kinase domain at their C-terminal ends, such as AtETR1 and AtERS1 from *A. thaliana*; subfamily II receptors have four transmembrane regions at their N-terminal ends and a diverged histidine kinase domain at their C-terminal ends, such as AtETR2, AtERS2, and AtEIN4 from *A. thaliana* (22).

Cu(I) ions are essential cofactors for ethylene binding in the plant ethylene receptors. The conservative amino acids C65 and H69 residing in the EBD of AtETR1 are concerned with binding of the copper cofactor (23). Ag(I) ions can preferentially bind to plant ethylene receptors but do not mediate ethylene signal transduction (23, 24). 1-Methylcyclopropene (1-MCP) competes with ethylene to bind plant ethylene receptors and acts as an ethylene-activity inhibitor (25).

The white button mushroom, Agaricus bisporus, is one of the most widely cultivated and consumed edible fungi worldwide. In previous studies, we found that the ethylene biosynthesis pathway of these button mushrooms is the ACC pathway; ethylene inhibits mushroom mycelial growth and primordium formation (4, 26) but promotes postharvest mushroom maturation and senescence and upregulates maturation- and senescence-related genes. Maturation- and senescence-related genes have several plant ethylene response elements in their promoter regions, and ethylene response elements such as W boxes, ethylene responsive elements (EREs), and dehydration responsive element/C-repeat (DRE/CRT) motifs can respond to ethylene regulation (27). However, how ethylene is perceived by the mushroom remains unknown.

Diverse hybrid histidine kinases are coded in fungal genomes, which are divided into 11 groups (28). Four hybrid histidine kinases are found in the button mushroom genome, belonging to fungal hybrid histidine kinase group II, group III, group VI, and group X. A protein (ID 201390 in JGI [https://jgi.doe.gov/]) in the *A. bisporus* var bisporus (H97) genome exhibits all domain structures of plant ethylene receptors but includes five transmembrane regions in the putative EBD, rather than the three found in AtETR1, as observed in group II; another protein (ID 143539) shows similar domain structures to the ID 201390 protein except that the GAF domain is absent, as observed in group VI (29). Both proteins are likely potential ethylene receptors. In this study, we investigated the ethylene-binding activities of these two putative ethylene receptors of *A. bisporus* and compared the ethylene response characteristics of the two putative ethylene receptor gene antisense transformants to those of their parent strains.

## RESULTS

### Amino acid sequence alignment of EBDs between the putative ethylene receptors of *A. bisporus* and the ethylene receptors of other species.

**FIG 1:**
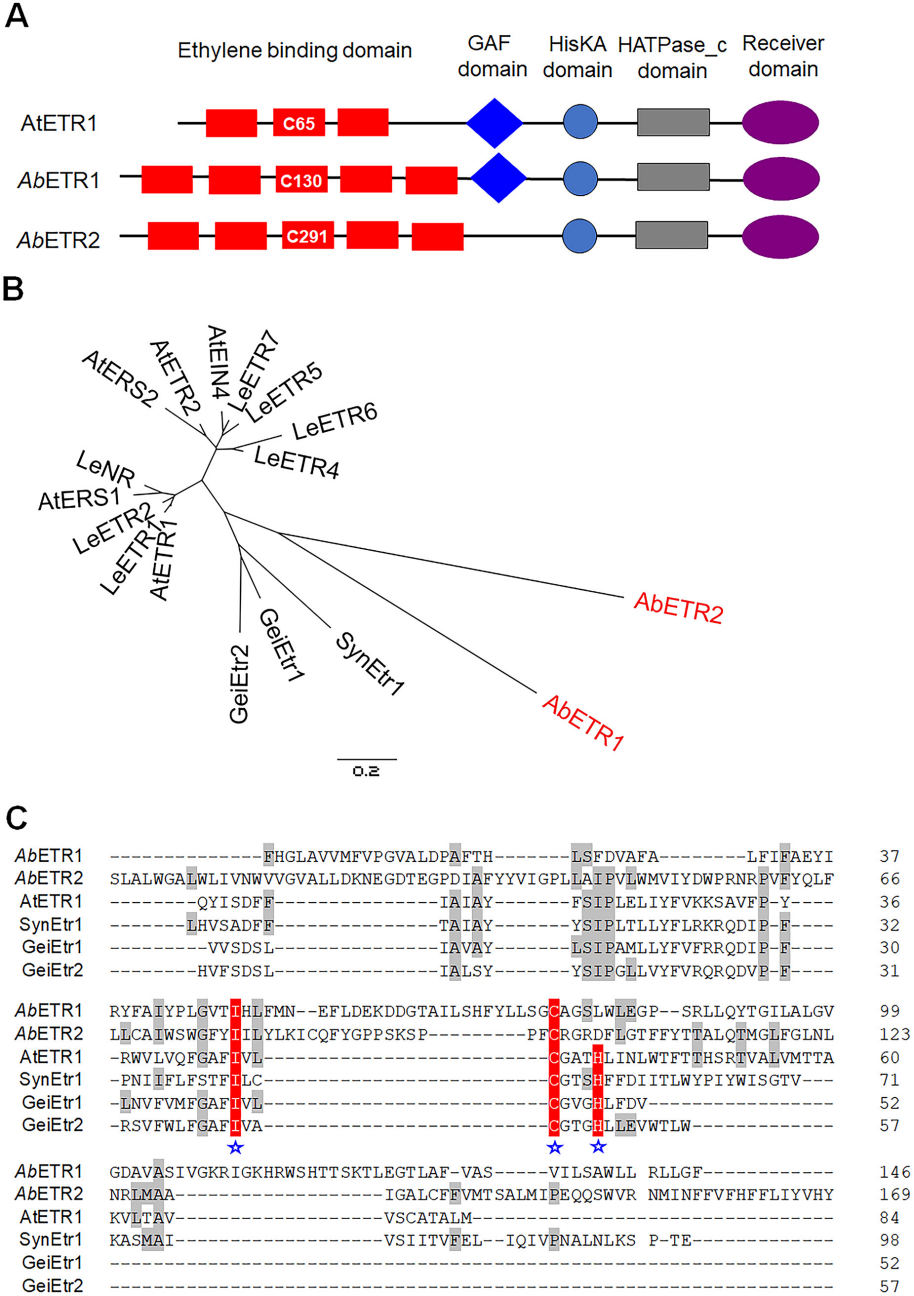
Structural comparison and amino acid sequence alignment of the ethylene binding helices in EBDs between the selected ethylene receptors. (A) Structural comparison of Arabidopsis thaliana ETR1 and the two putative ethylene receptors of *Agaricus bisporus*. The red boxes are transmembrane domains or ethylene binding domains. The essential cysteine residues to bind ethylene in the transmembrane domain are indicated. HisKA, His kinase A (phosphoacceptor) domain; HATPase_c, histidine kinase-like ATPases; receiver domain, cheY-homologous receiver domain; GAF domain, domain present in phytochromes and cGMP-specific phosphodiesterases. (B) Unrooted dendrogram for amino acid sequences of the ethylene binding and signaling helices in EBDs between the putative ethylene receptors of *A. bisporus* and the other elected ethylene receptors. (C) Amino acid sequence alignment of the ethylene binding and signaling helices in EBDs between the putative ethylene receptors of *A. bisporus* and the other elected ethylene receptors. The amino acid residues in *Ab*ETR1 and *Ab*ETR2 which are homologous to the other ethylene receptors are indicated with gray shading. The amino acids in EBD that were conserved and essential for ethylene binding are indicated by white letters on red background marked with blue asterisks. *Ab*ETR1 and *Ab*ETR2, the putative ethylene receptors of *A. bisporus* with JGI protein ID 201390 and 143539; AtEIN4, AtERS1, AtERS2, AtETR1, and AtETR2, the ethylene receptors of *A. thaliana* with the NCBI accession numbers NP_187108, NP_181626, NP_001323287, NP_176808, and XP_002883407, respectively; SynEtr1, the ethylene receptor of *Synechocystis* sp. PCC 6803 with the NCBI accession number NP_440714; GeiEtr1 and GeiEtr2, the ethylene receptors of *Geitlerinema* sp. PCC 7105 (14); LeETR1, LeETR2, LeETR4, LeETR5, LeETR6, LeETR7, and LeNR, the ethylene receptors of Lycopersicon esculentum with the NCBI accession numbers NP_001234149, NP_001234153, NP_001234205, NP_001234212, NP_001234150 (77), and NP_001233894, respectively.

The two potential ethylene receptors of the button mushroom contained a cysteine residue (Cys130 and Cys291) in transmembrane helix 3, as found in AtETR1, in which a cysteine residue (Cys65) residing in transmembrane helix 2 is an essential amino acid for ethylene binding (23) (Fig. 1A). We designated them *Ab*ETR1 (ID 201390) and *Ab*ETR2 (ID 143539). In addition, several predicted plant ethylene response elements existed in the promoter regions of the *AbETR1* and *AbETR2* genes, including one W box in the *AbETR1* promoter region and three DRE/CRT motifs in the *AbETR2* promoter region. The EBD amino acid sequences of the putative ethylene receptors of the mushroom were aligned with the ethylene receptors of *A. thaliana*, Lycopersicon esculentum, and cyanobacteria, and *Ab*ETR1 and *Ab*ETR2 and the cyanobacterium receptors formed a distinct subfamily (subfamily III) different from the other two subfamilies (subfamily I and subfamily II) of plant receptors (Fig. 1B). The EBD amino acid sequences of *Ab*ETR1 and *Ab*ETR2 harbored two out of three essential residues that have been reported to be involved in ethylene perception (17, 23) (Fig. 1C).

### Ethylene-binding activities of *Ab*ETR1 and *Ab*ETR2.

**FIG 2:**
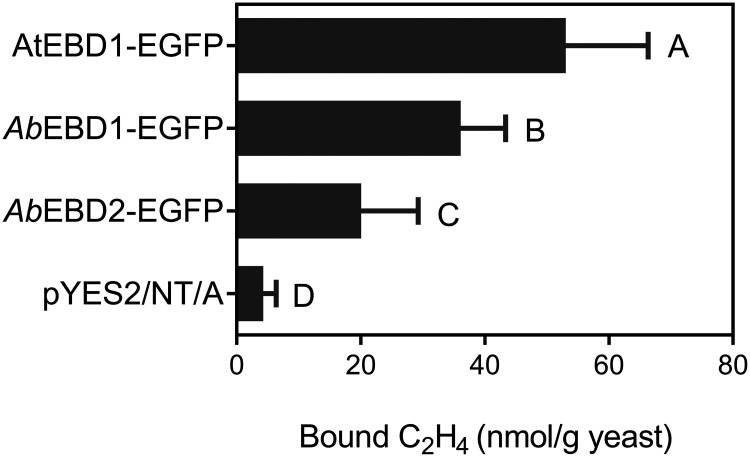
Ethylene bound by the yeast cells expressing the fusion protein of EGFP and the ethylene binding domain of the ethylene receptors from *Agaricus bisporus* and Arabidopsis thaliana. The yeast cell pellets were packaged in sealed glass bottles. The bottles were evacuated and injected with ethylene gas and then incubated at 22°C for 4 h. After ethylene gas not bound by cells was removed, the bottles were evacuated and metal-bathed at 65°C for 90 min to release the bound ethylene. The released ethylene gas was quantified by gas chromatography. *Ab*EBD1-EGFP, fusion protein of EGFP and the ethylene-binding domain *Ab*EBD1 from *A. bisporus* ethylene receptor *Ab*ETR1; *Ab*EBD2-EGFP, fusion protein of EGFP and the ethylene-binding domain *Ab*EBD2 from *A. bisporus* ethylene receptor *Ab*ETR2; AtEBD1-EGFP, fusion protein of ethylene binding domain AtEBD1 from *A. thaliana* ethylene receptor ETR1; pYES2/NT/A, unloaded plasmid. Data are expressed as the averages of nine samples ± the standard error (SE) from three independent experiments, and different capital letters indicate that the difference is very significant (*P* < 0.01) based on one-way analysis of variance (ANOVA).

The EBDs of AtETR1, *Ab*ETR1, and *Ab*ETR2 fused with enhanced green fluorescent protein (EGFP) were expressed in yeast (see Fig. S1 in the supplemental material), and the amount of ethylene bound by the yeast was assayed. Similar to AtETR1, both *Ab*ETR1 and *Ab*ETR2 had ethylene-binding activities; however, the amounts of ethylene binding were lower than that of AtETR1, while *Ab*ETR1 had a higher ethylene-binding capacity than *Ab*ETR2 (Fig. 2).

### Verification of the *A. bisporus* antisense *EBD* transformants.

To investigate the effects of the downregulation of the putative ethylene receptors on the biological characteristics of button mushroom, we constructed antisense *EBD* expression transformants. Two stable transformants were obtained and designated *Ab* as-ETR1-2 and *Ab* as-ETR2-17 after subculturing 5 times on complete yeast extract (CYM) (30) plates or CYM plates containing 40 μg mL^−1^ hygromycin. The *hph* genes and antisense *AbETR1* or antisense *AbETR2* were amplified from the two transformant genomes using PCR but were not amplified from their parent strain (wild type [WT]) (Fig. S2), and the PCR products were confirmed using sequencing.

**FIG 3:**
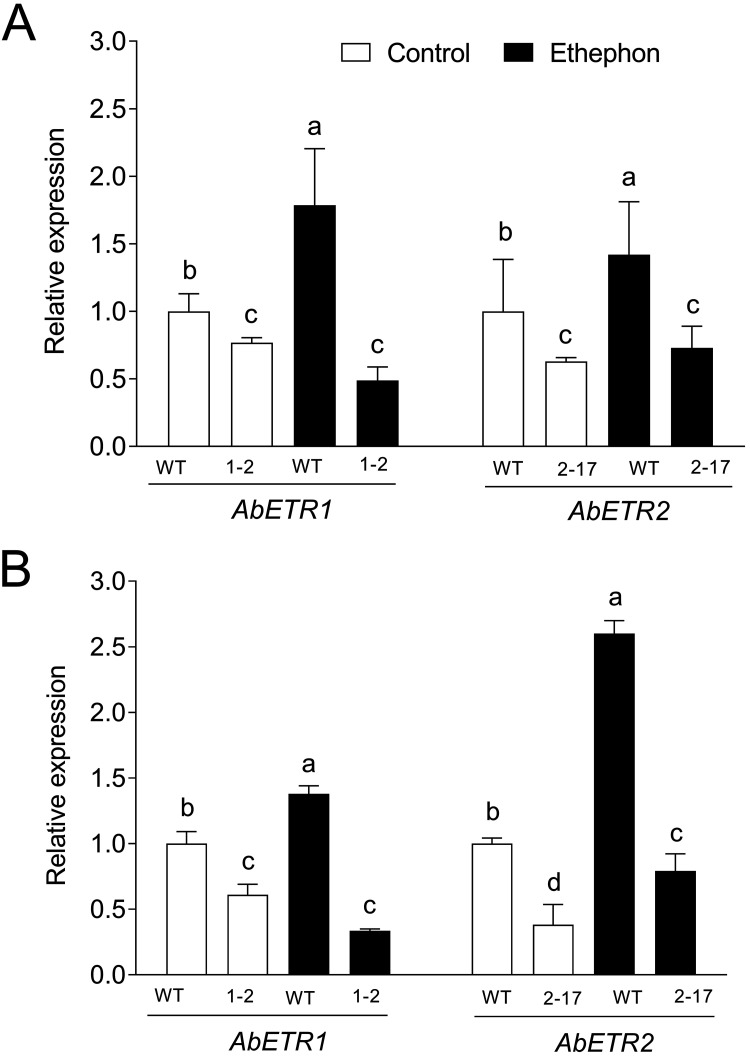
The expression level of the putative ethylene receptor genes in mycelia and postharvest fruiting bodies of *Agaricus bisporus* antisense *EBD* transformants and their parent strain As2796 with and without ethylene treatment. The mycelia grown on cellophanes were transferred to new plates containing the medium with or without 100 μM ethephon and cultivation continued for 24 h. After that, the mycelia were collected and used to extract total RNA for quantitative RT-PCR. The postharvest mushrooms were treated with 0.05% ethephon or water and then stored at 20°C. (A) Mycelia treated with ethephon for 24 h; (B) the postharvest fruiting bodies for storage for 1 h. WT, wild-type As2796; 1-2, *Ab* as-ETE1-2; 2-17, *Ab* as-ETR2-17. Data indicate the mean ± SE (*n* = 3) of three independent experiments. Data marked with different lowercase letters are statistically different at *P < *0.05 based on one-way ANOVA.

The *AbETR1* and *AbETR2* expression of *Ab* as-ETR1-2, *Ab* as-ETR2-17, and the WT in mycelia was determined by quantitative reverse transcriptase PCR (RT-PCR) with and without ethephon treatment. The mycelia were cultivated on nutrient-rich potato dextrose agar medium paved with cellophane at 25°C for 20 days. The cellophanes were transferred to new plates containing the medium with or without 100 μM ethephon and cultivation was continued for 24 h. The *AbETR1* and *AbETR2* expression of *Ab* as-ETR1-2 and *Ab* as-ETR2-17 was downregulated by 23% and 37%, respectively, compared to those of the WT without ethephon treatment; in contrast, their expression was downregulated in *Ab* as-ETR1-2 and *Ab* as-ETR2-17 but upregulated in the WT under treatment with ethephon for 24 h compared to those of the WT without ethephon treatment (Fig. 3A). Similar *AbETR1* and *AbETR2* expression was found for the postharvest fruiting bodies after storage for 1 h of *Ab* as-ETR1-2, *Ab* as-ETR2-17, and the WT (Fig. 3B).

### Mycelial growth rate and ethylene production of *A. bisporus* antisense *EBD* transformants.

**FIG 4:**
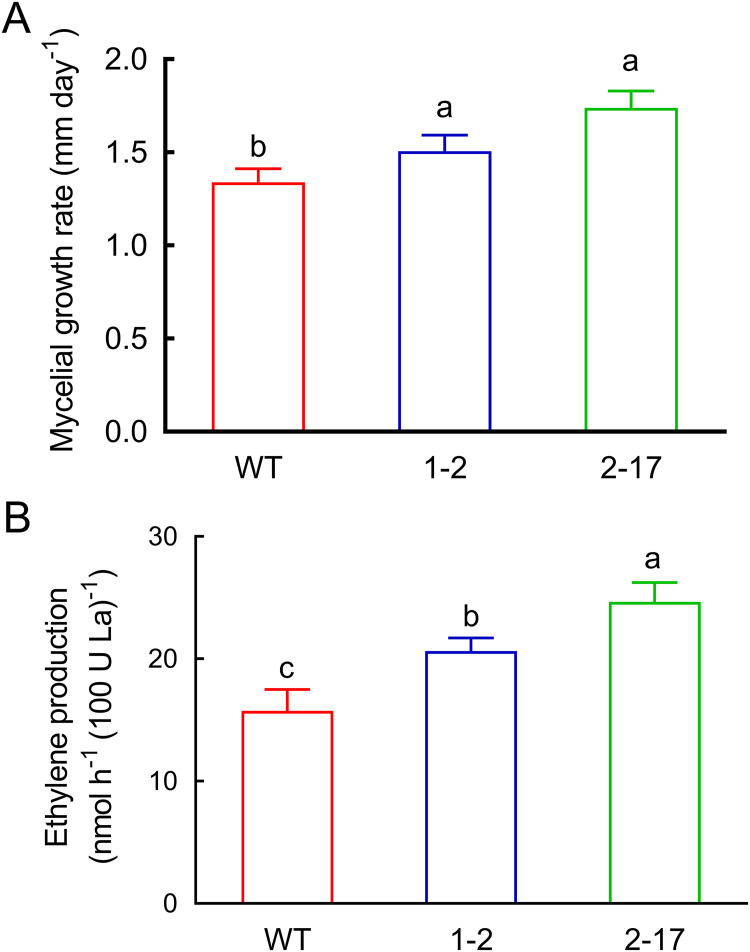
(A and B) Mycelial growth rate (A) and ethylene production (B) of *Agaricus bisporus* antisense *EBD* transformants and their parent strain growing in sterile mushroom compost tubes. The mycelial biomass of *A. bisporus* was represented by laccase activities. 1-2, *Ab* as-ETR1-2; 2-17, *Ab* as-ETR2-17; La, laccase; WT, wild-type As2796. Data indicate the mean ± SE (*n* = 3) of three independent experiments. Data marked with different lowercase letters are statistically different at *P < *0.05 based on one-way ANOVA.

The mycelial growth rates of *Ab* as-ETR1-2 and *Ab* as-ETR2-17 in sterile compost tubes were 12% and 30% higher, respectively, than those of the WT (Fig. 4A). Moreover, the ethylene production from the mycelia of *Ab* as-ETR1-2 and *Ab* as-ETR2-17 growing in sterile compost was 31% and 57% higher, respectively, than those of the WT, while that of *Ab* as-ETR2-17 was 19% higher than that of *Ab* as-ETR1-2 (Fig. 4B).

### Ethylene production and appearance quality characteristics of the postharvest fruiting bodies from the *A. bisporus* antisense *EBD* transformants.

**FIG 5:**
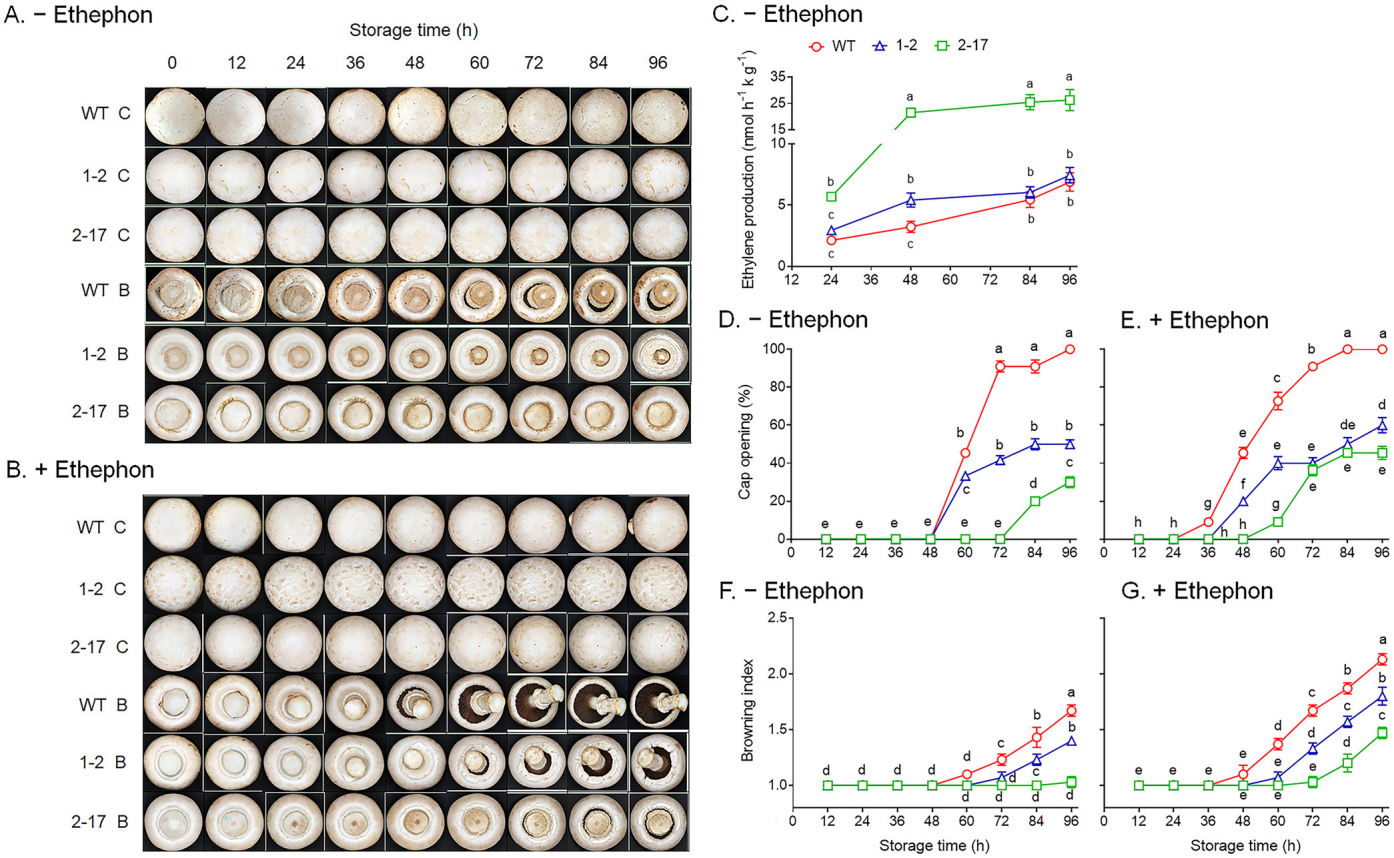
Changes in appearance characteristics and ethylene production of the postharvest fruiting bodies from *Agaricus bisporus* antisense *EBD* transformants and their parent strain during storage at 20°C for 96 h. (A) Fruiting bodies without ethephon treatment; (B) fruiting bodies with ethephon treatment; (C) ethylene production; (D) cap opening of fruiting bodies without ethephon treatment; (E) cap opening of fruiting bodies with ethephon treatment; (F) browning index of fruiting bodies without ethephon treatment; (G) browning index of fruiting bodies with ethephon treatment. C, cap; B, bottom; WT, wild-type As2796; 1-2, *Ab* as-ETR1-2; 2-17, *Ab* as-ETR2-17. The cap opening rate was determined by calculating the percentage of the number of veils opened to the total number of mushrooms. The browning index was obtained by counting the rate of browning area to cap surface and scoring from 1 to 5 points: 1, no browning on the cap surface; 2, the browning area does not exceed 1/4 of the cap surface; 3, the browning area occupies 1/4 to 1/2 of the cap surface; 4, the browning area exceeds 1/2 of the cap surface; 5, the cap surface is completely browned. Data indicate the mean ± SE (*n* = 3) of three independent experiments. Data marked with different lowercase letters are statistically different at *P < *0.05 based on one-way ANOVA.

Postharvest button mushrooms accumulate reactive oxygen species (ROS) to trigger the synthesis of ethylene. Endogenous and exogenous ethylene (such as ethephon) promotes the postharvest mushrooms’ maturation and senescence (27, 31). To examine the effect of endogenous and exogenous ethylene on the postharvest mushrooms’ maturation and senescence, the postharvest button mushrooms were immersed in 0.05% ethephon or water (control) for 2 min and then stored at 20°C after being wiped gently with gauze and air-dried. Without ethephon treatment, the ethylene production from the postharvest fruiting bodies of the two transformants increased from 24 h to 48 h and then remained stable during storage at 20°C. Ethylene production from the postharvest fruiting bodies of the WT increased from 24 h to 84 h and then remained stable. The ethylene production from the postharvest fruiting bodies of *Ab* as-ETR2-17 was higher than those of the WT and *Ab* as-ETR1-2 during storage (Fig. 5C). The cap opening and browning of the postharvest fruiting bodies from *Ab* as-ETR1-2 and *Ab* as-ETR2-17 were delayed and reduced compared to those of the WT during storage at 20°C for 96 h. The cap opening of the postharvest fruiting bodies from *Ab* as-ETR1-2 and the WT both took place from storage for 60 h, but subsequently, the cap opening rate of *Ab* as-ETR1-2 was found to be 27% to 54% lower than that of the WT. The cap opening of the postharvest fruiting bodies from *Ab* as-ETR2-17 took place from storage for 84 h, and subsequently, the cap opening rate was found to be 70% to 78% lower than that of the WT in the same period (Fig. 5A and D). The postharvest fruiting bodies from the WT browned rapidly after storage for 60 h, whereas those from *Ab* as-ETR1-2 started browning after storage for 72 h, and the degree of browning was lower than that of the postharvest fruiting bodies from the WT in the same period. The postharvest fruiting bodies from *Ab* as-ETR2-17 showed almost no browning during storage (Fig. 5A and F).

With ethephon treatment, the cap opening and browning of the postharvest fruiting bodies from *Ab* as-ETR1-2 and *Ab* as-ETR2-17 were also delayed and reduced compared to those from the WT during storage for 96 h. The caps of the postharvest fruiting bodies from the WT began to open after storage for 36 h, and subsequently, the cap opening rate increased rapidly; when stored for 84 h, all of the caps opened. The cap of the postharvest fruiting bodies from *Ab* as-ETR1-2 and *Ab* as-ETR2-17 began to open after storage for 48 h and 60 h, and subsequently, the cap opening rates were found to increase slowly. The cap opening rates of the postharvest fruiting bodies from *Ab* as-ETR1-2 and *Ab* as-ETR2-17 were only 60% and 45%, respectively, of that from the WT when stored for 96 h (Fig. 5B and E). The postharvest fruiting bodies from the WT browned rapidly after storage for 48 h, whereas those from *Ab* as-ETR1-2 and *Ab* as-ETR2-17 began to brown after storage for 60 h and 84 h, respectively, and their degrees of browning were lower than that of the postharvest fruiting bodies from the WT in the same period (Fig. 5B and G).

### Effects of ethephon on the expressions of the two ethylene receptor genes in the postharvest fruiting bodies of the *A. bisporus* antisense *EBD* transformants.

**FIG 6:**
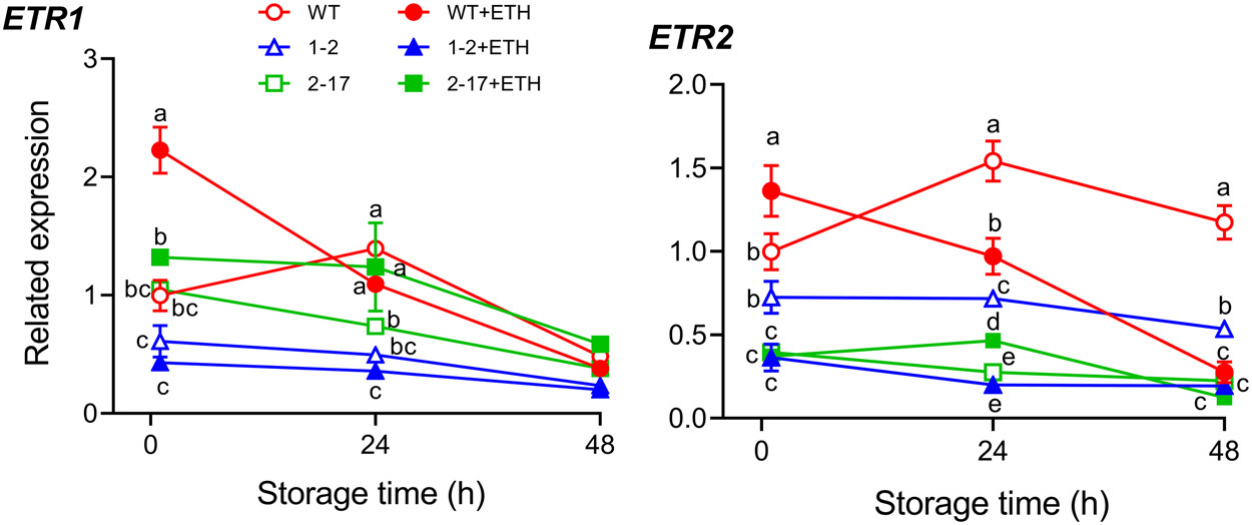
The expression of the two ethylene receptor genes in the postharvest fruiting bodies from *Agaricus bisporus* antisense *EBD* transformants and their parent after treatment without and with ethephon and storage at 20°C for 48 h. WT, wild-type As2796; WT+ETH, wild-type As2796 with ethephon treatment; 1-2, *Ab* as-ETR1-2; 1-2+ETH, *Ab* as-ETR1-2 with ethephon treatment; 2-17, *Ab* as-ETR2-17; 2-17+ETH, *Ab* as-ETR2-17 with ethephon treatment; *ETR1*, *AbETR1*; *ETR2*, *AbETR2*. Data indicate the mean ± SE (*n* = 3) of three independent experiments. Data marked with different lowercase letters are statistically different at *P < *0.05 for the same storage time based on one-way ANOVA.

The expressions of two ethylene receptor genes in the postharvest fruiting bodies were determined by quantitative RT-PCR duration storage for 48 h at 20°C. Increased expressions of *AbETR1* and *AbETR2* were observed in the WT treated with ethephon after 1 h compared to those of the WT without ethephon treatment, whereas, the expressions of *AbETR1* and *AbETR2* in *Ab* as-ETR1-2 and *Ab* as-ETR2-17 were lower than those in the WT in both treatment groups regardless of ethephon application. The expressions of two receptors all reduced in the same transformant under the same treatment and at the same time compared with that in the WT (Fig. 6).

### Effects of ethephon on the selected gene expression in the postharvest fruiting bodies of the *A. bisporus* antisense *EBD* transformants.

**FIG 7:**
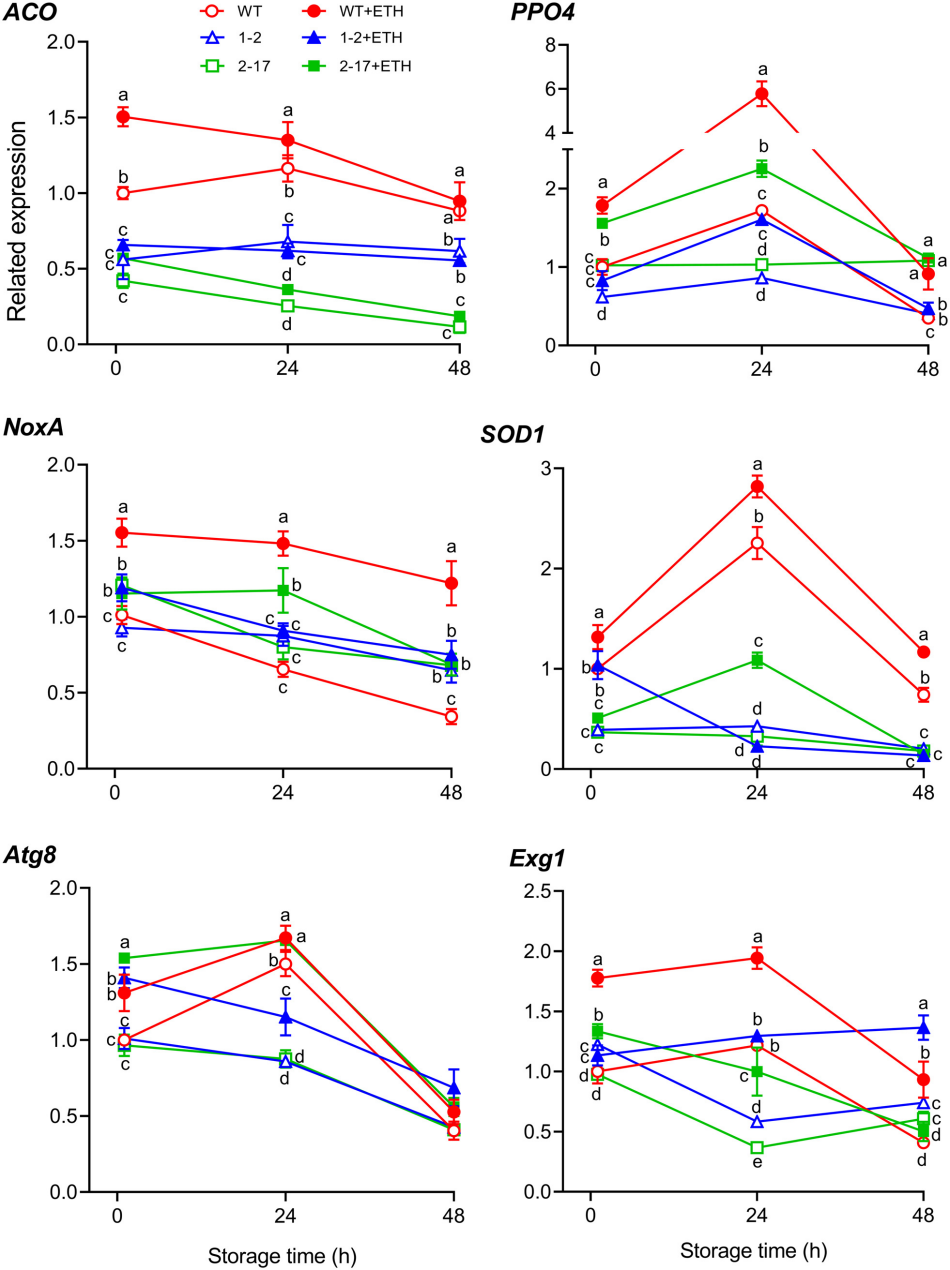
The expression of the selected genes related to postharvest fruiting body maturation and senescence in the postharvest fruiting bodies from *Agaricus bisporus* antisense *EBD* transformants and their parent after being treated without and with ethephon and stored at 20°C for 48 h. WT, wild-type As2796; WT+ETH, wild-type As2796 with ethephon treatment; 1-2, *Ab* as-ETR1-2; 1-2+ETH, *Ab* as-ETR1-2 with ethephon treatment; 2-17, *Ab* as-ETR2-17; 2-17+ETH, *Ab* as-ETR2-17 with ethephon treatment; *ACO*, ACC oxidase gene (GenBank accession number JQ314344); *Atg8*, autophagy-associated Atg8 gene (GenBank accession number XM_006458408); *Exg1*, exo-1,3-beta-glucanase gene (GenBank accession number XM_006454084; protein ID 196962); *NoxA*, NADPH oxidase A gene (GenBank accession number XM_006454614); *PPO4*, tyrosinase (PPO4) gene (GenBank accession number GU936494); *SOD1*, superoxide dismutase 1 gene (JGI protein ID 207393). Data indicate the mean ± SE (*n* = 3) of three independent experiments; Data marked with different lowercase letters were statistically different at *P < *0.05 for the same storage time based on one-way ANOVA.

Many genes are involved in postharvest button mushroom maturation and senescence (27, 31, 32), including respiratory burst oxidase or NADPH oxidase (Nox), antioxidant enzymes, ethylene synthase, browning enzymes, cell wall macromolecule-degrading enzymes, and autophagy-related enzymes (27, 33). The primary respiratory burst oxidase in fungi is NoxA (34). Throughout postharvest storage, Cu-Zn superoxide dismutase (SOD1) activity in button mushrooms is maintained at a high level and is the only antioxidant enzyme negatively correlated with the hardness of mushrooms (35, 36). The ethylene biosynthesis pathway of the button mushroom is the ACC pathway (4). ACC oxidase (ACO) is often the rate-limiting step of high ethylene production in ripening fruit (37). The button mushroom genome harbors only one copy of the gene encoding ACO (38). Polyphenol oxidases (PPOs) are major contributors to the browning of button mushrooms. The content of PPO4 is the highest among the six PPOs in button mushrooms (39). Glucan is the major fungal cell wall polysaccharide and is closely related to the firmness of button mushrooms during storage (40). β-glucan and exo-beta-1,3-glucanase 1 (Exg1) are the main polysaccharide and glucanase, respectively, in button mushrooms (41, 42). Autophagy occurs in the young and mature fruiting bodies of Pleurotus ostreatus (43) and results in fruit ripening (44, 45). Atg8 is highly conserved in eukaryotes and usually serves as a marker to monitor autophagosome formation (46). Thus, the six genes, *NoxA*, *SOD1*, *ACO*, *PPO4*, *Exg1*, and *Atg8*, were selected to investigate the expression pattern in the antisense *EBD* transformants. During storage at 20°C for 48 h, the expression of the six genes of the WT treated with ethephon were higher than those not treated with ethephon. Without ethephon treatment, the expressions of all genes of the two antisense *EBD* transformants were lower than those of the WT, except *NoxA*. With ethephon treatment, the expressions of all genes of the two antisense *EBD* transformants also were lower than those of the WT, except *Atg8* (Fig. 7).

## DISCUSSION

There is much evidence that higher fungi exhibit ethylene receptors with properties similar to those of plant ethylene receptors. Ethylene induces appressorium formation in *Colletotrichum*, but AgNO_3_ inhibits their induction by ethylene (47). 1-MCP eliminates the inhibitory effect of endogenous ethylene on aflatoxin synthesis by Aspergillus (48) and decelerates the maturation and senescence of the button mushroom after harvest (27). Therefore, higher fungi can be expected to exhibit ethylene receptors similar to those of plants.

The classic approach for ethylene receptor identification is to express the ethylene-binding domain in yeast and determine the amount of [^14^C] ethylene bound by the yeast cells or disrupted cells (12, 17, 19, 23, 49, 50). This method is sensitive and has a low threshold for ethylene quantitative determination; furthermore, the saturability of ethylene receptor binding to ethylene can be determined. However, this method requires radiolabeled ethylene and is difficult for most laboratories to perform. Gas chromatography (GC) with flame ionization detection (FID) and a photoionization detector (PID) achieves a much lower ethylene detection limit on the order of nL L^−1^ (51) and has been used to identify the ethylene released from the *Arabidopsis* ethylene receptor ETR1 protein expressed by yeast (19). However, the saturability of ethylene receptor binding to ethylene cannot be determined by GC. In this study, the amount of ethylene released from yeast expressing the EBDs of *Ab*ETR1 and *Ab*ETR2 was 67% and 43%, respectively, of the amount released from yeast expressing the ethylene-binding domain of the *Arabidopsis* ethylene receptor ETR1, indicating the high ethylene-binding activities of EBDs of *Ab*ETR1 and *Ab*ETR2.

The mycelial growth rates of *Ab* as-ETR1-2 and *Ab* as-ETR2-17 in sterilized compost were higher than that of their parent, and their ethylene production was also higher than that of their parent strain, suggesting that the two antisense ethylene receptor transformants had reduced sensitivity to ethylene’s inhibition of mycelial growth and to the regulation of ethylene synthesis. Accordingly, downregulated expressions of *AbETR1*, *AbETR2*, and most of the selected postharvest mushroom maturation- and senescence-related genes were observed in *Ab* as-ETR1-2 and *Ab* as-ETR2-17 regardless of ethylene application compared to those of their parent strain, showing insensitivity to ethylene’s regulation of gene expression.

The two ethylene receptors in *A. bisporus* belong to subfamily III, the same as the ethylene receptors in cyanobacteria. Deletion of only one receptor leads to a constitutive ethylene response in *Synechocystis*, indicating that the ethylene receptor negatively regulates the ethylene response, which is similar to that found in plants (12, 13, 52). Reduced expression of either of the two ethylene receptors resulted in not only decreased expression of the other ethylene receptor but also decreased ethylene sensitivity in the button mushroom. Thus, the two ethylene receptors in *A. bisporus* likely positively regulate the ethylene response, in contrast to that which occurs in plants and *Synechocystis*.

It is suggested that hormones must have a specific receptor (53). According to this proposition, the only fungal hormones are pheromones that have corresponding receptors that have been identified (54). Animal hormone binding proteins have been discovered in various fungi (55, 56); however, whether these binding proteins are hormone receptors has not been determined. 10-Oxo-trans-8-decanoic acid, 1-octen-3-ol, and hercynine are considered to be fungal hormones, all derived from *A. bisporus* (57), but their receptors have not been reported. The identification of the ethylene receptors of *A. bisporus* in this study strongly suggests that ethylene is a fungal hormone and fills the gaps in the knowledge pertaining to the absence of ethylene receptors in higher fungi.

Postharvest button mushrooms are highly perishable with a very short shelf life (58). Endogenous and exogenous ethylene speeds up the maturation and senescence and upregulates the maturation- and senescence-related genes of postharvest button mushrooms, the same as that of climacteric fruits (27, 59). The molecular mechanism is that the promoter regions of the maturation- and senescence-related genes in button mushrooms harbor ethylene response elements and can be induced by ethylene, similarly to plants (27). Applied individually or in combination with cooling, modified atmosphere packaging or 1-MCP treatment inhibits ethylene biosynthesis or ethylene-induced quality deterioration and prolongs shelf life (27, 60, 61). This study showed that reducing the expression of each of the two putative ethylene receptors of *A. bisporus* was an effective approach for preventing ethylene from binding and eliciting its action. In particular, reducing the expression of *AbETR2* was more effective than reducing the expression of *AbETR1*. *Ab*ETR2 did not have a GAF domain, the amount of ethylene bound to its EBD was lower than that of *Ab*ETR1, and the ethylene production of the mycelia and harvested fruiting bodies from *Ab* as-ETR2-17 was also higher than that from *Ab* as-ETR1-2, but the mycelial growth rate of *Ab* as-ETR2-17 was higher than that of *Ab* as-ETR1-2. The cap opening and browning of the harvested fruiting bodies from *Ab* as-ETR2-17 were prolonged and reduced compared to those from *Ab* as-ETR1-2. The reason for this may be that the reduced expression of *AbETR2* caused the strain to be less sensitive to ethylene than *AbETR1*.

In this study, we used ethephon instead of ethylene to investigate the effect of exogenous ethylene on the expression of the button mushroom ethylene receptors and on the mycelial growth and postharvest mushroom maturation and senescence of the antisense ethylene receptor transformants because ethylene gas may not be conveniently available to nonethylene laboratories for occasional use (62–64). Ethephon is a widely used chemical replacement for ethylene that is expediently added to media at concentrations of 10 nM (65) or 100 μM (27), sprayed at 25 to 100 ppm (173 nM to 692 nM) (66) or 1,000 mg L^−1^ (6.9 mM) (67), or used as an immersion solution for several minutes at a strength of 0.05% (3.4 mM) (27) or 2 mg/mL (13.8 mM) (68). Ethephon (2,4-dichlorophenoxyacetic acid) decomposes slowly to produce ethylene and simultaneously produces phosphate and chloride at a pH above 4.0 (64). Therefore, using ethephon as a replacement for ethylene treatment may not be appropriate for quantitative experiments accompanied by adverse effects with a low pH condition resulting from phosphate and chloride and a long ethylene response window (64).

Beet discs are placed in 100 mg L^−1^ (0.69 mM) (pH 3.1) ethephon, which increases betacyanin leakage from the beet root discs, while pigment leakage is not influenced by bubbling ethylene through a neutral medium containing the beet discs (69), implying that the strong acid produced by ethephon decomposition misdirects the physiological processes of ethylene. In contrast, when rice and watergrass seeds are maintained in liquid medium containing 1,000 mg L^−1^ (6.9 mM) ethephon, germination percentages are not significantly influenced until higher ethephon concentrations are used (70). Over a 3-day decomposition, the solution pH of ethephon (8.3 mM to 1,000 mM) in disodium hydrogen phosphate buffer (Na_2_HPO_3_, 5 mM) remains above 7.40 (63). An SH medium (comprising 2.0% glucose, 0.5% peptone, and 0.5% yeast extract) is used. When 0.01 mM to 1.0 mM ethephon with an initial pH value of 7 is used to cultivate Komagataeibacter xylinus for cellulose production, the pH value of the medium is slightly higher than that of the untreated control for the first 5 days, slightly lower than that of the control on days 6 to 12, and higher than that of the control for the 13th to 14th days of the 14-day culture period. Furthermore, the same concentration of phosphate-chloride does not inhibit bacterial growth and does not influence cellulose production or the final pH of cultures compared to those of the untreated control (71). This indicates that with the use of a normal concentration and time period, employing ethephon by adding it to the medium, or by spraying or immersing experimental materials in it for a short time, the adverse effects of the acid from ethephon degradation should not be a consideration.

In addition, the ethylene production reaches a maximum value over a 3-day decomposition of 100 μL of 8.3 mM to 1,000 mM ethephon in disodium hydrogen phosphate buffer (Na_2_HPO_3_, 5 mM). Ethylene was released rapidly on the first day, but the rate of release then slowed dramatically. The amount of ethylene released is 25% to 32% of the maximum value within the first 6 h and reaches 56% to 71% of the maximum value within the first day (63). Similar results are also observed in a buffer containing ethephon and tobacco leaf discs (72). Interestingly, treatment with 10 μL/L ethephon, 10 mM gibberellic acid (GA), or 4 mM indoleacetic acid (IAA) for 1 h generates similar depressed activities of catalase, peroxidase, and polyphenol oxidase in the excised pea stem tissue segments (73). Thus, when the treatment time is 1 day or more, the instability of ethylene produced by ethephon decomposition should have little effect on the experimental results.

In summary, we first demonstrated that the transmembrane regions of the two hybrid histidine kinases of *A. bisporus* had ethylene-binding activities, and the essential amino acids for ethylene binding were similar to those of *Arabidopsis* ETR1. Each of the two hybrid histidine kinase gene antisense RNA strains revealed reduced sensitivity to the ethylene inhibition of mycelial growth, regulation of ethylene synthesis, postharvest mushroom maturation and senescence, and expression of maturation- and senescence-related genes. To our knowledge, this is the first report on the biologically functional ethylene receptors in higher fungi, and their mode of action was different from that of plant and cyanobacterium ethylene receptors. Ethylene should be considered a novel fungal hormone. Reduced expression of ethylene receptors may facilitate the prevention of the maturation and senescence of postharvest button mushrooms.

## MATERIALS AND METHODS

### Strains and DNAs.

*A. bisporus* As2796 was provided by the Edible Fungi Research Institute of the Fujian Academy of Agricultural Sciences and was maintained and incubated on nutrient-rich potato dextrose agar medium (27). The plasmid pBHG-BCA1-gpd was kindly provided by Baogui Xie of Fujian Agriculture and Forestry University. The plasmid pBHG-BCA1-gpd harbored a hygromycin B phosphotransferase gene (*hph*), which was selected as a resistance marker. The plasmid pEGFP-C1 was purchased from Clontech (Mountain View, CA, USA), and Saccharomyces cerevisiae INVSC1 and the expression vector pYES2/NT/A were purchased from Shanghai Beinuo Biotechnology Co., Ltd. (Beinuo, Shanghai, China). The primers used for PCR amplification are listed in Table S1.

### Bioinformatics analysis.

Functional domain prediction for the two putative ethylene receptors of *A. bisporus* was performed by using the Simple Modular Architecture Research Tool (SMART) (http://smart.embl-heidelberg.de/). Multiple alignments of amino acid sequences were conducted with Clustal Omega (http://www.ebi.ac.uk/Tools/msa/clustalo/). The ethylene response elements in the promoter regions of the two putative ethylene receptors were predicted by using PlantCARE (http://bioinformatics.psb.ugent.be/webtools/plantcare/html/) (74) and PLACE (https://sogo.dna.affrc.go.jp/cgi-bin/sogo.cgi?lang=%20en=pj=640=action=page=page=newplace) (75).

### PCR for the cloning of the ethylene-binding domain genes.

Total RNA was extracted from the mycelia of *A. bisporus* grown in a petri dish and from *A. thaliana* leaves by using an RNAiso Plus total RNA extraction kit (TaKaRa, Dalian, China) according to the manufacturer’s instructions. Subsequently, cDNA was synthesized using the Maxima first-strand cDNA synthesis kit for RT-qPCR (no. K1641) (Thermo Fisher Scientific, Waltham, MA, USA). The ethylene-binding domains (EBDs) of *AbETR1*, *AbETR2*, and *AtETR1* were cloned by PCR using cDNA as a template with the primer pairs SGD-R and SGD-F, Type-R and Type-F, and AtETR1-F and AtETR1-R. The three EBDs were designated *Ab*EBD1, *Ab*EBD2, and AtEBD1. The EGFP gene expression cassette was amplified by PCR using the plasmid pEGFP-C1 as a template and EGFP-R and EGFP-F as primers. Fusion fragments of the EBDs and EGFP were produced by PCR using the obtained PCR products as templates with the primer pairs EGFP-F and SGD-OV-EGFP, EGFP-F and Type-OV-EGFP, and EGFP-F and AtETR1-OV-EGFP. The antisense EBDs of *AbETR1* and *AbETR2* were obtained by PCR using *A. bisporus* cDNA as a template with the primer pairs SGD-SspeI and SGD-Xba I and Type-SpeI and Type-XbaI.

### Construction of EBD expression vectors and yeast transformation.

The fusion fragments of the EBDs and EGFP were digested with BamHI and EcoRI and ligated into the yeast expression vector pYES2/NT/A to generate the fusion protein expression vectors. The fusion protein expression vectors were then transformed into yeast INVSC1 using the LiAc/SS carrier DNA/PEG method (76).

### Protein expression and cell ethylene-binding test.

Transformed yeast INVSC1 colonies were cultivated in SC-Ura medium containing 2% glucose at 30°C and 220 rpm until the optical density at 600 nm (OD_600_) reached 0.4. The cultures were centrifuged at 4,000 rpm for 5 min to harvest the cells. The cells were suspended in SC-Ura medium with 2% galactose and 1% raffinose instead of glucose and cultivated for 24 h. The expressed fusion proteins were examined using a laser confocal fluorescence microscope (DMi8; Leica Microsystems, Germany). Whole-cell ethylene binding was tested using gas chromatography. The details of the test to determine the concentration of ethylene bound by the yeast cells are as follows. The cell pellets (0.6 g fresh weight) were transferred to 5-mL rubber-sealed glass bottles (Taidian, Yiwu, China). After the application of vacuum, each bottle was injected with 3,000 μL L^−1^ of ethylene gas and incubated at 22°C for 4 h. During incubation, the bottles were gently shaken so the cells would be fully exposed to the ethylene gas. After the binding was complete, the lids were opened, the bottles were ventilated for 10 min, and the cells were transferred to new bottles. The bottles were sealed and subjected to vacuum application and then placed in a 65°C metal bath for 90 min to release the bound ethylene in the cells. The gas in the headspace of the bottles was sampled with a gas-tight syringe, and the ethylene concentrations were measured with a gas chromatograph (GC-2010 Plus, Shimadzu, Japan). The chromatographic conditions were as follows: a GDX-502 capillary column (2 m by 3 mm inside diameter [i.d.]), column temperature of 60°C; hydrogen ion flame detector (FID), detector temperature of 150°C; carrier gas N_2_, flow rate of 20 mL min^−1^; combustion gas H_2_, flow rate of 40 mL min^−1^; and injector temperature of 110°C. The bound ethylene of the EBDs was expressed as nmol ethylene g^−1^ fresh weight yeast cells.

### Construction of antisense EBD expression vectors and transformation.

The *as-EBD*s of *AbETR1* and *AbETR2* were processed by using SpeI and XbaI and ligated into the pBHG-BCA1-gpd plasmid, generating the antisense *EBD* expression vectors pBHG-asEBD1-gpd and pBHG-asEBD2-gpd. The *as-EBD* expression vectors were transformed into *A. bisporus* using the *Agrobacterium*-mediated fruit body tissue culture transformation method (77).

### Mycelial growth rate tests.

To detect the mycelial growth of the *A. bisporus* As2796 and antisense *EBD* transformants, the mycelia were inoculated into test tubes containing sterilized compost and cultivated at 25°C for 30 days. Mycelial elongation was measured at intervals of 4 days to calculate the mycelial growth rate.

### Postharvest fruiting body storage.

*A. bisporus* As2796 and antisense *EBD* transformants were routinely cultivated. Harvested mushrooms were treated with water as a control and 0.05% ethephon and then stored at 20 ± 1°C and an rH of 85 to 90% (27). The stored mushrooms were sampled at intervals of 12 h for quality measurement and liquid nitrogen preservation. The cap opening rate and browning index were tested as described previously (27, 78). The cap opening rate was defined as the percentage of the number of veils opened to the total number of mushrooms. The browning index was the rate of browning area to cap surface scoring from 1 to 5 points.

### Ethylene production determination.

Ethylene production was determined by gas chromatography as described previously (26, 27) when the mycelia colonized 2/3 of the sterilized-compost test tubes, and the postharvest fruiting bodies were stored for 24 h to 96 h. Ethylene production of the mycelia was expressed as nmol h^−1^ per 100 U of laccase. Ethylene production of the fruiting bodies was expressed as nmol ethylene h^−1 ^kg^−1^ fresh weight.

### Laccase activity assays.

The mycelial biomass of *A. bisporus* in compost is positively correlated with the activity of laccase; therefore, the measurement of laccase activity in culture compost allows the mycelial biomass of *A. bisporus* to be easily characterized (79). The laccase activity of the *A. bisporus* culture compost was assayed by a method involving 2,2′-azobis(3-ethylbenzothiazole-6-sulfonaic acid) (ABTS) as the substrate (80).

### Detection of gene expression in mycelia and postharvest fruiting bodies by quantitative RT-PCR.

Gene expression in the mycelia and postharvest fruiting bodies was tested by quantitative RT-PCR as previously described (27).

### Data availability.

All data generated during this study are included in full in this paper and its supplemental information files.
