# Supplementary material for: Two Hybrid Histidine Kinases Involved in the Ethylene Regulation of the Mycelial Growth and Postharvest Fruiting Body Maturation and Senescence of Agaricus bisporus
Source: Microbiol Spectr. 2022 Sep 20;10(5):e02411-22. doi: 10.1128/spectrum.02411-22 (PMC9603746; doi:10.1128/spectrum.02411-22)
Supplement: Supplemental file 1 — Table S1, Fig. S1, Fig. S2. Download spectrum.02411-22-s0001.pdf, PDF file, 0.5 MB [file spectrum.02411-22-s0001.pdf]

Supplemental Material

Table S1 Primers used in this study

Primers [1]	Nucleotide sequence (5′–3′)	Application
SGD-R	GGATCCATGCAGGCGAATGCGCTG	Cloning of *Ab*ETR1 EBD
SGD-F	GAATCCAAGAAGCCGCAATAGC	
Type-R	GGATCCAGTTTAGCACTTTGGGGGGC	Cloning of *Ab*ETR2 EBD
Type-F	GTAGTGCACATATATAAGGAAGAAC	
EGFP-R	ATGGTGAGCAAGGGCGAG	Cloning of EGFP gene expression cassette
EGFP-F	GAATTCCTTGTACAGCTCGTCCATGC	
SGD-OV-EGFP	GCTTCTTGGATTCATGGTGAGCAAGGGCGAG	Fusion of *Ab*ETR1 EBD and EGFP
Type-OV-EGFP	GCCTGTACACATGGTGAGCAAGGGCG	Fusion of *Ab*ETR2 EBD and EGFP
AtETR1-R	ATGGAAGTCTGCAATTGTATTG	Cloning of AtETR1 EBD
AtETR1-F	CTCAGCAGCTTTATTTTTCAA	
AtETR1-OV-EGFP	AGCTGCTGAGATGGTGAGCAAGGGCGAG	Fusion of AtETR1 EBD and EGFP
SGD-Spe I	GGACTAGTGAATCCAAGAAGCCGCAA	Cloning of *Ab*ETR1 asEBD
SGD-Xba I	GCTCTAGATTTCACGGGCTTGCAGT	
Type-SpeI	GGACTAGTGTAGTGCACATATATAAGGAAGAA	Cloning of *Ab*ETR2 asEBD
Type-XbaI	GCTCTAGAAGTTTAGCACTTTGGGGG	
Hph-F	CTTCTGCGGGCGATTTGTG	Transformant verification for gene *hph*
Hph-R	TCGTTATGTTTATCGGCACTTT	
SGD-t35s-f	GAATCCAAGAAGCCGCAATAG	Transformant verification for AbETR1 as*EBD*
SGD-t35s-r	CCCTTATCTGGGAACTACTCAC	
Type-t35s-f	GTAGTGCACATATATAAGGAAGAAG	Transformant verification for *Ab*ETR2 as*EBD*
Type-t35s-r	CCCTTATCTGGGAACTACTCAC	
Ef1a-F	AACAAGGTTCCCCTGGTGAC	qRT-PCR for housekeeping gene
Ef1a-R	CGGGTGATTGAGGACGATG	
SGD-F1	CGTATGGTGTGGAATGGGC	qRT-PCR for *AbETR1*
SGD-R1	TCAGCAAACGCCTGTCCA	
Type-F1	TTTGGGGGGCACTCTGGC	qRT-PCR for *AbETR2*
Type R1	TGGGATAGCCAACAGAGG	
Exg-F	GCTTGTCTTGTCCCCCCTT	qRT-PCR for *Exg*
Exg-R	CGCGCAAAATCTTCTTCTTCCGTG	
PPO4-F	GCAGGACGCAGGATAACCTTGT	qRT-PCR for *PPO4*

PPO4-R	GGACGACCTTGGACTCGGATTC	
SOD1-F	GCTCAAGCCTGGTCCTCTCAAG	qRT-PCR for *SOD1*
SOD1-R	TGATACGGACATGACGCACGAC	
NoxA-F	TTGCCGTGACTCGCCTTCCT	qRT-PCR for *NoxA*
NoxA-R	GGTCGTATTCCGCTCCAGTGTC	
Atg8-F	TAGCCTCCGTCTTGTCCACC	qRT-PCR for *Atg8*
Atg8-R	TGTCCGTTCTATCTGCCTTTTC	
ACO-F	ATGGAACCCACCCAGAACC	qRT-PCR for *ACO*
ACO-R	TTGCGATGAGGCGAAGAT	

[1] *Atg8*, autophagy-associated Atg8 gene；*Exg1*, exo-1,3-beta-glucanase gene; *NoxA*, NADPH oxidase A gene; *PPO4*, tyrosinase (PPO4) gene; *SOD1*, superoxide dismutase 1 gene.

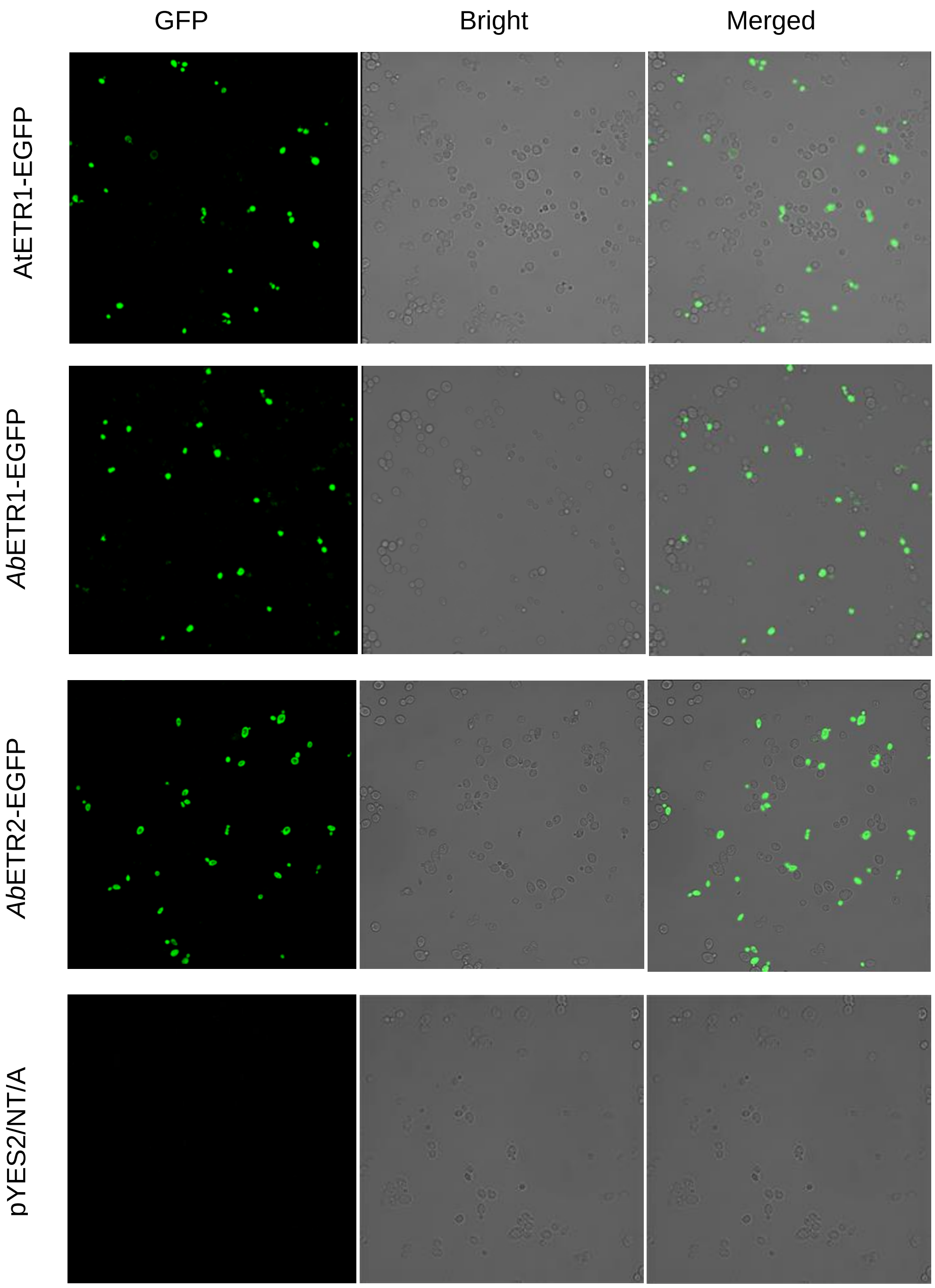

Fig S1 Fluorescence microscopic observation of the yeast cells expressing the fusion protein of EGFP and the ethylene binding domain of the ethylene receptors from *Agaricus bisporus* and *Arabidopsis thaliana*. *Ab*EBD1-EGFP, fusion protein of EGFP and the ethylene-binding domain *Ab*EBD1 from *A. bisporus* ethylene receptor *Ab*ETR1; *Ab*EBD2-EGFP, fusion protein of EGFP and the ethylene-binding domain *Ab*EBD2 from *A.*

bisporus ethylene receptor *Ab*ETR2; AtEBD1-EGFP, fusion protein of ethylene binding domain AtEBD1 from *A. thaliana* ethylene receptor ETR1; pYES2/NT/A, unloaded plasmid.

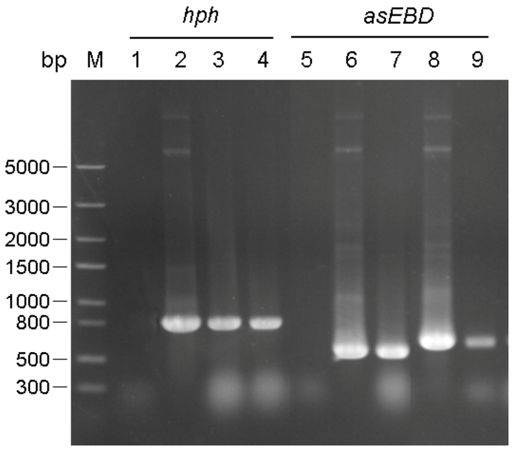

Fig S2 PCR verification of *hph* and as*EBD* genes in *Agaricus bisporus* as*EBD*s transformants. M, Trans5K DNA Ladder; 1, As2796; 2, pBHg-asEBD1-gpd; 3, *Ab* as-ETR1-2; 4, *Ab* as-ETR2-17; 5, As2796; 6, pBHg-asEBD1-gpd; 7, *Ab* as-ETR1-2; 8, pBHg-asEBD2-gpd; 9, *Ab* as-ETR2-17.
